# Qualitative interviews with adults with Classic Galactosemia and their caregivers: disease burden and challenges with daily living

**DOI:** 10.1186/s13023-022-02287-9

**Published:** 2022-03-28

**Authors:** Jason A. Randall, Carolyn Sutter, Stella Wang, Evan Bailey, Lydia Raither, Riccardo Perfetti, Shoshana Shendelman, Claire Burbridge

**Affiliations:** 1 Clinical Outcomes Solutions, Unit 68 Basepoint, Shearway Business Park, Shearway Road, Folkestone, Kent, CT19 4RH UK; 2 Clinical Outcomes Solutions, Chicago, IL USA; 3 Applied Therapeutics, New York, NY USA

**Keywords:** Classic Galactosemia, GALT deficiency, Qualitative interviews, Patient experience, Caregiver experience, Health-related quality of life, HRQoL, Burden of illness

## Abstract

**Background:**

Classic Galactosemia is a rare, autosomal recessive disease in which galactose is not metabolized properly due to severe deficiency/absence of the galactose-1-phosphate uridylyltransferase (GALT) enzyme, converting to an aberrant and toxic metabolite, galactitol. Newborn screening and timely galactose-restricted diet can resolve acute symptoms and decrease fatalities. However, despite this, significant chronic, progressive morbidities remain which have a real impact upon daily life. To better understand the burden of disease, 20 in-depth qualitative interviews were undertaken with adult patients (n = 12), and their caregivers (n = 8), enrolled in the ACTION-Galactosemia trial, part of a clinical program designed to investigate the safety and efficacy of AT-007 (govorestat) in reducing toxic galactitol and long-term clinical outcomes in Classic Galactosemia.

**Results:**

Interviews revealed the substantial burden of Classic Galactosemia on patients and families. Most adults were not able to live independently, and all required support with day-to-day activities. Short- and long-term memory difficulties and tremors were identified as the most frequently experienced and challenging symptoms. Other difficulties such as fine motor skills and slow/slurred speech contribute to the significant impact on daily activities, affecting ability to communicate and interact with others. Symptoms were first noticed in early childhood and worsened with age. Classic Galactosemia impacted all areas of daily functioning and quality of life, leading to social isolation, anxiety, anger/frustration and depression. This demonstrates the significant burden of disease and challenges associated with Classic Galactosemia.

**Conclusions:**

The impact on both patients and caregivers underscores the severity of the unmet medical need and the importance of pharmacological intervention to halt or prevent disease progression. Any treatment that could reduce symptoms or slow functional decline would ease the burden of this condition on patients and caregivers.

## Background

Galactosemia is a rare autosomal recessive condition with mutations in the enzymes involved in the Le Loir pathway of galactose metabolism. Classic Galactosemia, also known as Type I Galactosemia, is a result of galactose-1-phosphate uridylyltransferase (*GALT*) gene mutation, and is the most common form of Galactosemia, occurring in 1/16,000 to 1/60,000 births worldwide [1, 2].

As of 2004, newborn screening programs for Galactosemia are mandatory in the United States (US) and most western countries, which has been critical in limiting fatalities due to the disease [3]. The only currently available disease management strategy is life-long dietary galactose restriction [1]. If infants with Classic Galactosemia are not treated promptly with a low-galactose diet, life-threatening complications appear within a few days after birth. Affected infants typically develop feeding difficulties, lethargy, failure to thrive, jaundice, liver damage, and abnormal bleeding. Other serious complications of this condition can include overwhelming sepsis and shock as well as cerebral edema and seizures [4, 5]. The advent of newborn screening and prompt initiation of a galactose-restricted diet has significantly decreased acute complications in the newborn period. However, this has not prevented long-term complications in patients with Galactosemia, who still face significant progressive worsening of disease symptoms throughout life, despite strict adherence to the galactose-restricted diet.

Dietary modifications are not sufficient to prevent long-term complications because the human body naturally produces galactose at levels 10 times higher than those resulting from residual galactose in the restricted diet, and patients lack the enzymes necessary to metabolize this galactose normally. Thus, even with strict dietary modifications, galactose produced by the body builds up and is converted to the toxic and aberrant metabolite galactitol by the enzyme aldose reductase [4, 5]. Accumulation of toxic galactitol appears to be the primary and driving factor in acute and long-term complications. Although other contributing factors have been postulated, including impaired glycosylation, altered gene expression, oxidative stress, and endoplasmic reticulum stress [6, 7], none of them has been conclusively demonstrated to have a critical role in Classic Galactosemia. Thus, even patients with early diagnosis and strict dietary adherence can experience early onset and progression of the symptoms of Classic Galactosemia as well as long-term complications [4, 5].

Longer-term complications include delayed development, cataracts, speech difficulties, and intellectual disability. This can develop into neurologic and central nervous system abnormalities such as tremor, ataxia, leukodystrophy, and difficulties with spatial and visual perception [2, 8–10].

Most adults with Galactosemia have an IQ under 85 and are not able to live independently [1, 11]. Adult patients may also experience anxiety and/or depression, and females with Classic Galactosemia have premature ovarian insufficiency along with its consequences of delayed pubertal onset and need for hormonal replacement therapy. Living with the debilitating symptoms and long-term consequences of Galactosemia yields a heavy burden on patients’ and their families’ lives [1, 11]. To ensure a patient centered approach in drug development, it is important to fully understand the lived experience and burden of the condition from the patient perspective so that this can be appropriately evaluated as part of the development of new treatments for Classic Galactosemia.

Despite this, there is a lack of qualitative research in the area. Thus, there is a need to better understand the experience of Classic Galactosemia in patients’ daily lives and the impact this has on their ability to live independently. Therefore, the current study was conducted to explore the patient experience of Classic Galactosemia from the patient and caregiver perspective and provide critical data towards understanding of the unmet need for treatment in Classic Galactosemia. This approach follows regulatory guidance for capturing the patient voice as an integral part of patient-focused drug development and collecting comprehensive and representative data [12, 13].

## Results

### Participant characteristics

All except 1 of the eligible patients participated in an interview. The one patient who did not participate was due to time restrictions and other commitments. Therefore, a total of 20 participants (12 patients and 8 caregivers who were caring for 9 of the patients in this study) completed interviews for this study. Caregivers were not diagnosed with Classic Galactosemia, they discussed their experience of caring for someone (the patient) who had Classic Galactosemia. All eight caregivers had a patient taking part in the study, one participant was a caregiver to two of the patients interviewed for this study. Demographic and patient clinical health data is presented in Table 1.

**Table 1:** Participant demographic and clinical health characteristics

Demographic characteristic	Patient total (n = 12)	Caregiver total (n = 8)
Gender		
Female	5 (41.7%)	6 (75.0%)
Male	7 (58.3%)	2 (25.0%)
Age^a^		
Mean (SD)	29.3 (10.24)	55.9 (9.52)
Median	24.0	55.5
Q1, Q3	21.5, 38.5	47.0, 64.5
Min, Max	19, 46	45, 68
Race		
White	12 (100%)	8 (100%)
Ethnicity		
Not Hispanic/Latino	12 (100%)	8 (100%)
Has caregiver		
No	4 (33.3%)	–
Yes	8 (66.7%)	–
Education		
High school diploma (or GED)	–	1 (12.5%)
Some college or certification program	–	1 (12.5%)
College or university degree (2- or 4-year)	–	5 (62.5%)
Graduate degree	–	1 (12.5%)
Work Status		
Employed full-time (≥ 40 h per week)	–	2 (25.0%)
Employed part-time (< 40 h per week)	–	1 (12.5%)
Homemaker	–	2 (25.0%)
Retired	–	3 (37.5%)
Relationship to patient		
Parent	–	8 (100%)
Live with Patient		
Yes	–	5 (62.5%)
No	–	3 (37.5%)
GALT enzyme activity^b^		
0.0 nmol/h/mg of hemoglobin	12	–
*GALT* gene mutations		
Q188R (homozygous)	8	–
Q188R, D98N	1	–
Q188R, Other	1	–
Q188R, K285N	1	–
Q188R, L195P	1	–

*GALT* galactose-1-phosphate uridylyltransferase; *N* number of subjects in the population; *Q1* 25th Percentile; *Q3* 75th Percentile; *SD* standard deviation

^a^Patient age is reported based on year of birth provided via the clinical trial data. Age was calculated using this year and the date of programming (September 2021)

^b^Patients’ GALT enzyme activity was collected during their enrolment in the ACTION-Galactosemia clinical trial, between August 2019 to August 2021

Four patients lived semi-independently, three of whom reported not having a formal caregiver to participate in an interview. However, it was evident from the interviews that they still received significant support from family members in day-to-day activities.

### Conceptual model of Classic Galactosemia

**Fig. 1:**
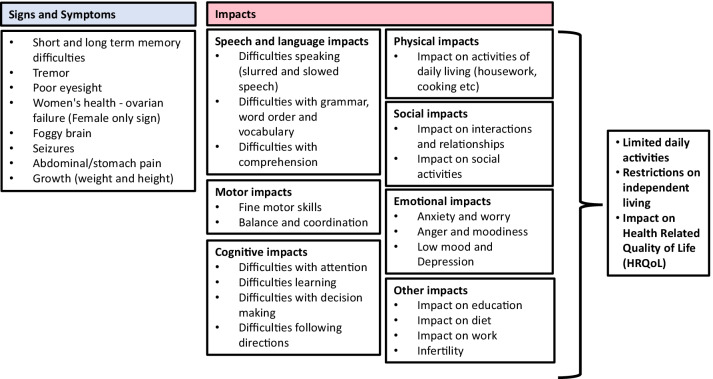
Patient-led Conceptual model of Classic Galactosemia. *Note* Some caregivers also discussed how their child experienced jaundice as a newborn. As this is a sign that only occurs as a newborn, it has not been included in the conceptual model

The interviews provided rich data on the lived experience of Classic Galactosemia, highlighting the burden Classic Galactosemia has on day-to-day life and the challenges it creates for living independently. Several signs and symptoms were identified, which were reported to impact upon the individual in many ways (emotional, social, motor, cognitive, physical, speech and language, other), all of which ultimately had a detrimental effect upon the patients’ health-related quality of life (HRQoL). Saturation was met, with no new concepts being identified in the final round of interviews. Therefore, it was assumed that further interviews were not needed as all key concepts had been identified and additional interviews would only identify information on topics already covered. A conceptual model was developed to illustrate the lived experience of Classic Galactosemia, based upon these qualitative insights, see Fig. 1.

### Symptoms

**Fig. 2:**
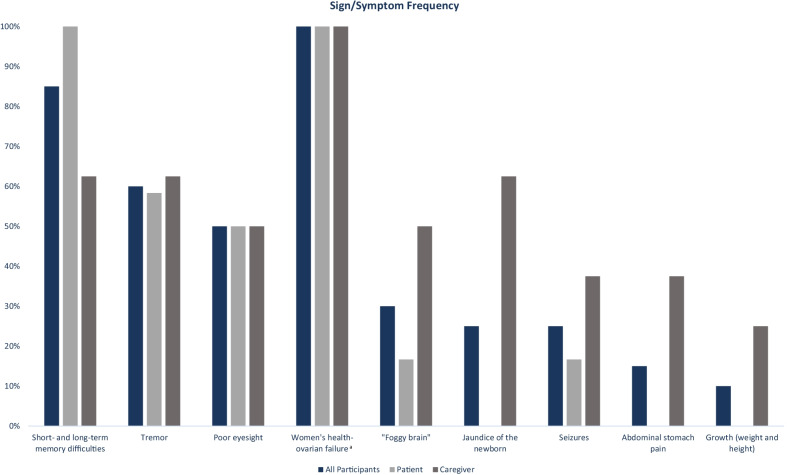
Frequency graphs for Classic Galactosemia signs/symptoms. ^a^The percentage of participants who discussed women’s health-ovarian failure was calculated as a percentage of the number of female patients (n = 5) and caregivers of female patients (n = 4)

As can been seen in the conceptual model, patients and caregivers spoke about a variety of symptoms. The 3 most frequently discussed were short- and long-term memory difficulties (n = 12/12 patients and 5/8 caregivers), tremors (n = 7/12 patients and 5/8 caregivers), and poor eyesight (n = 6/12 patients and 4/8 caregivers). These signs and symptoms of Classic Galactosemia were not only the most prevalent but were also those that had the most impact on the patients’ day-to-day lives, their activities of daily living (ADLs) and their ability to live independently. In addition, all female patients (n = 5) and caregivers of female patients (n = 4) described the patients’ experiences related to ovarian failure, and the impacts this had for infertility and hormonal maturation as well as severe emotional impacts for patient and caregiver. Figure 2 presents the percentages for how many patients discussed each symptom.

Memory difficulties created many challenges for patients. Individuals experienced difficulties communicating, as they needed longer to process information in conversations and respond to questions, which impacted their interactions with others and ability to socialize. One caregiver explained that her daughter has a recall delay and requires additional time to answer questions “*When she started school was when we kind of started noticing I think they call it relay delays, so… if [they] would ask a question with a normal 3–5 s turnaround, hers would be 10–15 s*” Caregiver-03. During childhood this created challenges with learning and education, and in adulthood resulted in issues with key ADLs, functioning at work (if they were able to hold a job), and ultimately their ability to live independently. For example, one patient described the impact of his memory difficulties at work and how he would often ask people to repeat instructions, *“[if] my boss tells me to do something in a specific order, and I might have to ask another time, like, once or twice, two more times at the most”* Patient-07. This patient needed to rely on his boss to help him manage his short-term memory challenges. Other patients and caregivers also discussed patients needing support to deal with memory difficulties in their day-to-day lives, which was required by most patients. One patient also explained that his memory difficulties have impacts for his health, as he sometimes forgets to take his medication “*I almost forgot to take the medication, and then I forgot to take the medication*” Patient-11.

Tremors also led to an extensive burden on patients’ day-to-day lives, particularly with fine motor activities such as handwriting, which led to challenges during education and work as well as personal care such as brushing teeth. A wide range of activities were impacted, which affected the patient’s ability to live independently, and so tremors were felt to be important. Tremors also caused patients to stand out from others, which was felt as a child at school and as an adult when at work or socializing, as highlighted by one patient who explained: *“I remember in school the kids would notice it [the tremor], and ask me about it… [as an adult] other people look at me funny, and uh, I don’t want that. I just explain to them that I have a tremor and they understand, but. So social, socially I think it has impacted me”* Patient-05. As adults the tremors could be misinterpreted, with others thinking the individual was dealing with addiction (alcohol or drugs) which heightened the social impacts and resulted in further emotional effects. This had a severe impact, as one patient highlighted when he explained: “*if I could have a treatment that fixed one thing, I would want it to fix that [the tremor]”* Patient-09.

Ovarian failure, which was identified in female patients in puberty, resulted in considerable issues for these patients. During puberty this caused patients to stand out even more from their peers since they would not sexually mature and required hormone therapies. This was important to both patients and caregivers, since patients already stood out and had difficulties with social interactions, additional physically visible differences in appearance contributed additional burden. In addition, ovarian failure also resulted in infertility, which created an enormous emotional burden not only on the patients directly, but also upon the caregiver, who struggled not only with acceptance of a long-term progressive disease in their child, but also the loss of future grandchildren or generational continuity. For female patients, ovarian failure appeared to be one of the most important aspects of their Classic Galactosemia and had a substantial emotional impact often leading to feelings of depression and anxiety.

### Impacts

**Fig. 3:**
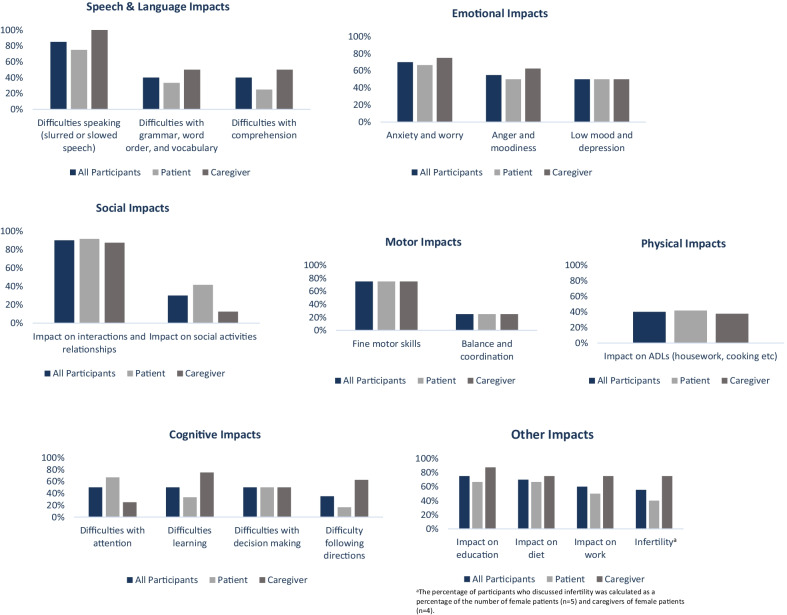
Frequency graphs for Classic Galactosemia impacts

As can be seen in the conceptual model, the impacts of Classic Galactosemia were substantial and upon all key aspects of HRQoL. Some impacts were more obviously related to specific symptoms, such as cognitive, speech and language, and motor impacts, and others were broader impacts affected by many or all symptoms together, such as physical, social, and emotional well-being. Classic Galactosemia was shown to limit ADLs, restrict ability to live and function independently, and impair overall HRQoL (Fig. 3).

Although all patients discussed an impact on their ADLs, these were often managed by having a caregiver. Therefore, beyond this, the impact most frequently discussed was impacts related to social aspects of life, such as impacts on social interactions and relationships (n = 11/12 patients and 7/8 caregivers) that led to feelings of social isolation and were particularly important to patients as was reported to have a substantial impact on their day-to-day life. Patients and caregivers related difficulties with social interactions to Classic Galactosemia as a whole and to specific symptoms such as tremors and cognitive difficulties (such as short- and long-term memory difficulties and foggy brain) as well as slowed and slurred speech. For example, one caregiver of a non-verbal patient explained that he struggled interacting due to being non-verbal: *“He doesn’t really have friends in the sense that people have friends, you know”* Caregiver-06. Caregivers highlighted that the types of social interactions expected of an individual the same age as the patient were not possible. This affected the patient’s ability to undertake day-to-day activities, function at school/work, and live independently, but also had an emotional impact: *“I think part of that is me not really wanting to put myself out there because I do feel like I don’t fit in, so why would I want to go into this into situations where I'm inviting that”* Patient-09. Although some patients can generally function on their own in terms of day-to-day activities and self-care, they may also feel isolated due to the social impacts of Classic Galactosemia.

Individuals also reported that Classic Galactosemia impacted their speech and language, which was associated with cognitive symptoms. Aspects of speech and language ranged from speech articulation (enunciation of words) to receptive and expressive language (ability to understand what is spoken to them and communicate thoughts). Difficulty speaking was described by n = 9/12 patients and 8/8 caregivers. Most patients and caregivers described difficulties with slowed speech and slowed speech response (receptive and expressive language), although many mentioned slurred speech (articulation). For some this can lead to challenges in communication that may require additional strategies, as one caregiver explained: *“we did sign language in the beginning, you know, and by now, we as this family understand him, I understand him the best but most of the family can kind of understand him pretty well”* Caregiver-06. For other patients their speech was extremely impacted to the point of being nonverbal. Across all levels, these speech problems had an impact on patients’ education, often requiring additional services such as speech therapy during school: *“I just remember struggling so much in class and having to be pulled out to a different class, a different classroom setting to work on, you know, my wordings and my articulations of certain words like the letter R. And just, I remember not really understanding a lot in school”* Patient-08. Difficulties with speaking and communicating were also linked to struggles with social interactions and feelings of isolation and frustration for patients since they could not easily be understood, and their difficulties impacted the way others perceived them. For example, 1 caregiver described: *“I think, sometimes, when he speaks people have a tendency to not take him very seriously, because he has a difficult time communicating”* Caregiver-07.

### Patient journey

Overall, most signs and symptoms of Classic Galactosemia were reported as first being noticed by the patients and caregivers in early childhood, except for ovarian failure which generally began around puberty for female patients. Although there is variation in the patient journey, typically, early symptoms were jaundice of the newborn, and growth issues. As the patient got older, other symptoms, such as tremor and cognition issues, were noticed. As time progressed these signs and symptoms typically appeared to worsen and had more of an impact on patients’ lives.

Caregivers also discussed the services and evaluations used by patients during childhood and into adulthood. Services and evaluations frequently described as being used during childhood were speech and language therapy (discussed by n = 7/8 caregivers), occupational therapy (n = 5/8 caregivers), and services for learning differences (n = 5/8 caregivers). Other services each reported as being used were mental health services, physical therapy, and attention deficit disorder/attention deficit hyperactivity disorder (ADD/ADHD) services. In comparison, the most frequent service described as being used by patients now (as an adult) were mental health services (n = 4/8 caregivers). Other services reported as currently being used during adulthood, each by only one caregiver, were: services for learning differences, physical therapy, ADD/ADHD services, vocational services, and life coach. It was also reported that most patients saw multiple specialists throughout their life; most frequently a geneticist and nutritionist/dietitian, each discussed by 7/8 caregivers. One caregiver also reported that her daughter saw a pediatric infertility specialist, and another caregiver explained that her son has seen a podiatrist because he needed aides to help with his walking due to his Classic Galactosemia.

When looking at the identified signs, symptoms, and impacts experienced by patients overall there were no clear differences by gender (except for ovarian failure, which is only experienced by female participants). In addition, although the numbers were low there was no difference by genetic subtype or by GALT enzyme activity (all subjects had 0.0 nmol/h/mg GALT enzyme activity).

### Independent living

The direct impact upon the patient was particularly evident for those who lived semi-independently in close proximity to family but did not have formal caregivers to support them in their day-to-day needs. Those with more severe symptoms not only lived with a caregiver but relied on the caregiver to perform functions that they struggled with or could no longer do, including ADLs. In these cases, a lot of the impact was felt on the caregiver. This highlights the substantial burden of Classic Galactosemia on patients’ lives and the impact this has on their ability to live independently. Classic Galactosemia thus had a wide-ranging impact upon both the patient and their family.

Patients and caregivers talked about the potential for, or current reality of, the impact of Classic Galactosemia on the patients’ ability to live independently. There are multiple ways that independent living was affected. For example, one caregiver explained her daughter needs reminders to take care of her personal hygiene because she will often forget *“she’ll just… not think that she needs to go have a shower, or that sort of thing. It’s always been something that’s been… a slight issue. She’s always had to be encouraged to take care of her personal hygiene basically”* Caregiver-10. Another stated: “*he's not going to be able to drive or live independently, or go to college, or have a job*” Caregvier-06. Most patients were unable to live independently and relied on a caregiver, a few were able to manage their symptoms and difficulties, with support, and were able to engage in work/some form of education program and live semi-independently. However, those living semi-independently still described the substantial support they needed from their caregiver or others to do this, and the fundamental difficulties Classic Galactosemia caused. For example, one caregiver explained that she still provides support for her son’s healthcare “*I’m the one who took him [to doctor’s appointments]; finding the right health care resources, as he got to be an adult; because he doesn’t, well, he struggles with processing and how to find the right resources; so, as an adult, I had to help him navigate through the health care system”* Caregiver-07.

Caregivers also highlighted the impact of caring for a child with Classic Galactosemia on their own lives, describing the fear and worry they experience related to the patients’ Classic Galactosemia and concern over who will provide support to the patient if they are no longer around to act as a caregiver. Caregivers also described the impact on their lives in terms of needing additional time and resources to support the patient. The most frequently discussed caregiver impacts were related to the emotional burden (n = 6/8) and the impact on the caregiver’s time and resources (n = 4/8) related to caring for someone with Classic Galactosemia.

## Discussion

The findings from this analysis of qualitative data from patient and caregiver interviews revealed that Classic Galactosemia has a substantial impact on the lives of the patients, their caregivers and their families. This is depicted within the conceptual model that was developed to illustrate the lived experience of Classic Galactosemia, based upon the patient and caregiver voices in this study. Classic Galactosemia is associated with a high burden of disease consisting of symptoms spanning across emotional, social, motor, cognitive, physical, speech and language, and impacting core aspects of life such as education, work, social interactions and ADLs.

In line with previous research, the current study identified tremors, cognitive impairments (difficulties with short- and long-term memory), ovarian failure, and difficulties with independent living as key symptoms and impacts of Classic Galactosemia [1, 2, 8, 11, 14]. Some signs and symptoms were described as beginning soon after birth or in early childhood, no matter the timing of patient diagnosis. The patient journey was variable and not all patients experience the same symptoms and impacts of the condition. This is consistent with previous research with siblings with Classic Galactosemia, which indicates that siblings diagnosed with Classic Galactosemia can experience a different disease journey [15]. Similar to prior survey research with patients with Classic Galactosemia, findings indicate cognitive impairments as well as internalizing problems (anxiety and depression) are common and often severe for adult patients [9, 10]. However, the current study has built on these learnings by adding greater depth and understanding of the lived experience from the patients and caregivers directly.

There was a range of experience and symptom severity for patients with Classic Galactosemia. Most patients interviewed were living at home with their caregiver and family, which is consistent with previous clinical literature [11]. However, this study also included some patients who could live semi-independently, and even those still described challenges related to daily living, and support they required at home or at work. Findings from these qualitative interviews suggest all patients will require some degree of support for the rest of their lives, ranging from support in daily decision making to constant care. Caregiver interviews also highlighted the clear burden and impact of providing support to patients with Classic Galactosemia, whether they lived with the patient or not. Given that the signs, symptoms, and impacts of Classic Galactosemia are known to worsen as the condition progresses, patients will need increasing amounts of support as they age leading to further burden for their families.

### Limitations

The current study was undertaken with English speaking US-based White American patients only, which may limit representativeness of the findings. Thus, although significant cultural differences in the lived experience of Classic Galactosemia are not anticipated, it was not possible to confirm the absence of cultural differences. As participants were recruited though the Applied Therapeutics clinical trial the sample was restricted to those who engaged in that trial. All who had been or were currently enrolled in the trial were approached, and all except 1 participant took part, meaning that the interview sample was highly representative of the clinical trial population. Medical information on patients was not collected in this study. Therefore, it is not possible to confirm how the full range of severity of Classic Galactosemia is represented clinically. While genetic data indicate all patients in this study have a Q188R allele, with 8 patients homozygous, all subjects have GALT enzyme activity of 0.0 nmol/h/mg making potential differences in genetics less likely to impact the observed phenotype. Indeed, the qualitative data suggest that it is highly likely this interview study included patients representing the full spectrum of adult Classic Galactosemia severity. Future research should replicate this study with a larger, demographically diverse sample to confirm the current findings are representative comprehensively capture the full patient experience.

## Conclusion

The qualitative data from this study demonstrate the significant burden of disease and challenges associated with Classic Galactosemia across all core aspects of life. The impact on both patients and caregivers underscores the severity of the unmet medical need and the importance of pharmacological intervention.

## Methods

### Interviews

In-depth qualitative interviews were conducted with Classic Galactosemia patients, and their caregivers, who were currently taking part in/had completed the Part D Extension portion of the ACTION-Galactosemia clinical trial exploring the safety and pharmacokinetics of AT-007 (govorestat). All those taking part were invited for the interview.

ACTION-Galactosemia clinical trial is a Phase 1–2 Dose-Escalating, 6-Part Study to Evaluate the Safety and Pharmacokinetics of Single and Multiple Doses of AT-007 (govorestat) in Healthy Adult Subjects and Adult Subjects with Classic Galactosemia (CG) or GALK-deficient Galactosemia. Part D and Part D Extension involved subjects who had a Classic Galactosemia diagnosis confirmed by evidence of absent or significantly decreased (< 1%) GALT activity in their red blood cells and by historical record of diagnosis of GALT deficiency (medical record or gene analysis report or written confirmation from healthcare professional), who have urine galactitol > 100 mmol/mol creatinine, and have no other significant health problems unrelated to Classic Galactosemia. To be eligible, patients also had to be male or non-pregnant, non-lactating female adults between the ages of 18 and 65 years. Patients were recruited for participation in the clinical trial via a selection of clinical sites across the US. No additional eligibility criteria were applied for this interview study other than a willingness to participate in the study and being sufficiently fluent in English to take part in an interview.

Subjects in the clinical trial were entered into Part D Extension after approximately 1 month in the Part D treatment arm of the study (during which they received placebo or active drug) or they may have entered Part D Extension without participating in Part D. Part D Extension involved treatment or placebo for up to 3 months. Some subjects had completed Part D Extension over a year prior, or were no more than two months into participating in Part D Extension.

Participants were recruited into this interview study by the clinical trial staff who shared information about the interviews and obtained informed consent. For patients and caregivers who provided consent, contact details were provided to Clinical Outcomes Solutions, an independent research company, who contacted the participants to schedule and conduct their interview. The interviews were conducted with participants either as they entered, during, or after completion of the Part D Extension.

All interviews were conducted in the US using an online phone system due to the impact of coronavirus-19 (COVID-19). Interviews with patients and their caregivers were conducted separately. Interviews and analyses were conducted by trained qualitative researchers, independent from Applied Therapeutics and the clinical trial.

A semi-structured discussion guide was used to explore the signs, symptoms, and impacts of Classic Galactosemia, to understand the burden of living with Classic Galactosemia for the patient, their caregivers and families, and to understand the impact on ability of the patient to live independently.

Interviews began with some brief initial questions to establish rapport and open the conversation, followed by a series of questions to spontaneously identify signs, symptoms, and impacts, and then focused questions to explore topics of interest including probes to explore relevant issues not previously mentioned. Caregivers were also asked to provide some background information related to patients’ medical history and specific services and evaluations the patient may have used, to aid the understanding of the patient’s journey. Interviews lasted approximately 60 min and were conducted by trained qualitative researchers. All interviews were recorded and transcribed verbatim. No therapeutic interventions or treatments were administered as part of this study.

All study documents for this study were approved by an independent review board as part of the main clinical trial documents. The study was conducted in compliance with Good Clinical Practice guidelines, including the International Conference on Harmonization Guidelines, and was consistent with the most recent version of the Declaration of Helsinki [16]. All applicable local laws and regulatory requirements were followed throughout the study.

### Analysis

Transcripts of the interviews were entered into NVivo 10 (updated to NVivo 1.0 during analysis), a software package designed to facilitate the storage, coding, and analysis of qualitative data. They were coded using thematic analysis to identify any themes, patterns, or features of interest within the data.

Saturation analysis was undertaken by dividing the study sample into 4 equal groups based on the chronological order in which they were interviewed. If a concept was discussed spontaneously or endorsed after probing, by at least 1 participant within a group, a check mark was added to the corresponding square. Saturation was considered met when no new themes or descriptions of concepts were identified in the final round of interviews.

## Data Availability

The datasets generated and/or analysed during the current study are not publicly available due the sensitive nature of the questions asked in this study but are available from the corresponding author on reasonable request.

## Abbreviations

ADD/ADHD: Attention deficit disorder/attention deficit hyperactivity disorder
ADLs: Activities of daily living
GALT: Galactose-1-phosphate uridylyltransferase
COVID-19: Coronavirus disease 2019
HRQoL: Health-related quality of life
US: United States
